# Relaxation processes in silicon heterojunction solar cells probed via noise spectroscopy

**DOI:** 10.1038/s41598-021-92866-w

**Published:** 2021-06-24

**Authors:** Kevin Davenport, C. T. Trinh, Mark Hayward, Klaus Lips, Andrey Rogachev

**Affiliations:** 1 Department of Physics and Astronomy, University of Utah, Salt Lake City, UT USA; 2 Competence Centre Photovoltaics Berlin (PVcomB), Helmholtz-Zentrum Berlin für Materialien und Energie GmbH (HZB), Berlin, Germany; 3 Department Spins in Energy Materials and Quantum Information Science (ASPIN), Helmholtz-Zentrum Berlin für Materialien und Energie GmbH (HZB), Berlin, Germany

**Keywords:** Applied physics, Condensed-matter physics, Electronics, photonics and device physics, Techniques and instrumentation, Energy science and technology

## Abstract

We have employed state-of-the-art cross-correlation noise spectroscopy (CCNS) to study carrier dynamics in silicon heterojunction solar cells (SHJ SCs). These cells were composed of a light absorbing *n*-doped monocrystalline silicon wafer contacted by passivating layers of *i*-*a*-Si:H and doped *a*-Si:H selective contact layers. Using CCNS, we are able to resolve and characterize four separate noise contributions: (1) shot noise with Fano factor close to unity due to holes tunneling through the np-junction, (2) a 1/*f* term connected to local potential fluctuations of charges trapped in a-Si:H defects, (3) generation-recombination noise with a time constant between 30 and 50 μs and attributed to recombination of holes at the interface between the ITO and *n-a*-Si:H window layer, and (4) a low-frequency generation-recombination term observed below 100 K which we assign to thermal emission over the ITO/*ni*-*a*-Si:H interface barrier. These results not only indicate that CCNS is capable of reveling otherwise undetectable relaxation process in SHJ SCs and other multi-layer devices, but also that the technique has a spatial selectivity allowing for the identification of the layer or interface where these processes are taking place.

## Introduction

Recent advances in the design and fabrication of silicon heterojunction solar cells (SHJ SCs) have boosted their operational parameters to record high values, exceeding 750 mV^1^ for open-circuit voltage, $V_{{OC}}$, and 26% efficiency^2^ and are the important ingredient of silicon/perovskite tandem solar cells that achieved the recently reported 29.15% record^3^ In the tandem solar cells, the charge-selective contacts for c-Si are *n*- and *p-*doped *hydrogenated amorphous silicon* (*a*-Si:H) which can be grown by the low-temperature process of plasma-enhanced chemical vapor deposition, PECVD. Direct contact between c-Si and doped *a*-Si:H results in interface defects which are identified as silicon dangling bond defects and traps states of the conduction and valence band tail of the a-Si:H^4–6^. This leads to additional recombination which reduces the V_OC_. To mediate this problem, a very thin layer of intrinsic hydrogenated amorphous silicon (*i-a*-Si:H) is added on one or both sides of the c-Si wafer to provide a better interface passivation^7–9^.

The desired optimal performance of an SHJ SC imposes a series of strict requirements on the *a*-Si:H layer. First, it must be very thin so to avoid series resistance and absorption losses. Second, it has to be grown and/or hydrogenated at a moderately high temperature with as little as possible doping to avoid a high concentration of defects but with high enough doping to establish a large shift of the Fermi level^8,10,11^. Furthermore, partial epitaxial growth has to be avoided since this also deteriorates interface properties^12^. The realization of the importance of these (and many other) processes would not have been possible without the detailed structural and electronic characterization of the *a*-Si:H thin layers and interfaces. In some cases, very specialized methods such as near-UV photoelectron spectroscopy^4^, surface photo-voltage measurements^6^, electron paramagnetic resonance^13,14^, and real-time spectroscopic ellipsometry^15^ have been used. However, one might expect that due to chemical and physical interactions, the properties of a layer alter when it becomes part of an active solar cell. Hence, further progress in the field, in particular reaching recently predicted 30% efficiency^16^, requires development of complimentary and specialized tools that can identify and characterize the performance-limiting elements in fabricated solar cells despite their complex structure (multi-layers stack plus electrodes).

In this paper, we use current-noise spectroscopy to detect and characterize different electronic relaxation processes in high-efficiency SHJ SCs. One well-known advantage of this method comes from the ability to resolve processes that occur on different time scales. The noise spectra reveal fingerprints of very specific electronic processes that conventional electrical characterization techniques are often not sensitive to. We argue here that current-noise spectroscopy also has a less obvious *spatial* selectivity, namely that it tends to magnify a contribution from the most resistive elements of the stacked layers.

Noise spectroscopy analyzes fluctuations of a signal from its equilibrium or steady-state value and is widely used for the characterization of defects and electronic relaxation processes in semiconducting devices^17–19^. It has been used in the past to study both doped and undoped *a*-Si:H^20^, *a*-Si:H -based transistors^21^, light-induced metastable changes in *a*-Si:H (Staebler-Wronski effect)^22^, and more recently to evaluate defect states in crystalline solar cells^23^. In all these works, however, the focus has been exclusively on the low frequency $1/f$ contribution to noise.

The technical advantage of our work comes from employment of cross-correlation current noise spectroscopy (CCNS)^24^, which provides two to four orders of magnitude improvement in the sensitivity and bandwidth of the measurements, giving access to regions of the noise spectrum which are typically hidden below the input noise floor. It is also very suitable for semiconductor devices with planar structure and high capacitance such as a typical photovoltaic cell. Using this technique, we were recently able to resolve the frequency-independent *shot* noise contribution in fluorescent^25^ and multi-layered phosphoresecent^26^ organic light emitting diodes (OLEDs). In addition, analysis of the magnetic field dependence of the *generation-recombination* noise term^21^ in fluorescent OLEDs helped us to identify the microscopic mechanism of the so-called organic magnetoresistance (OMAR) effect.

### Device structure and methods

**Figure 1:**
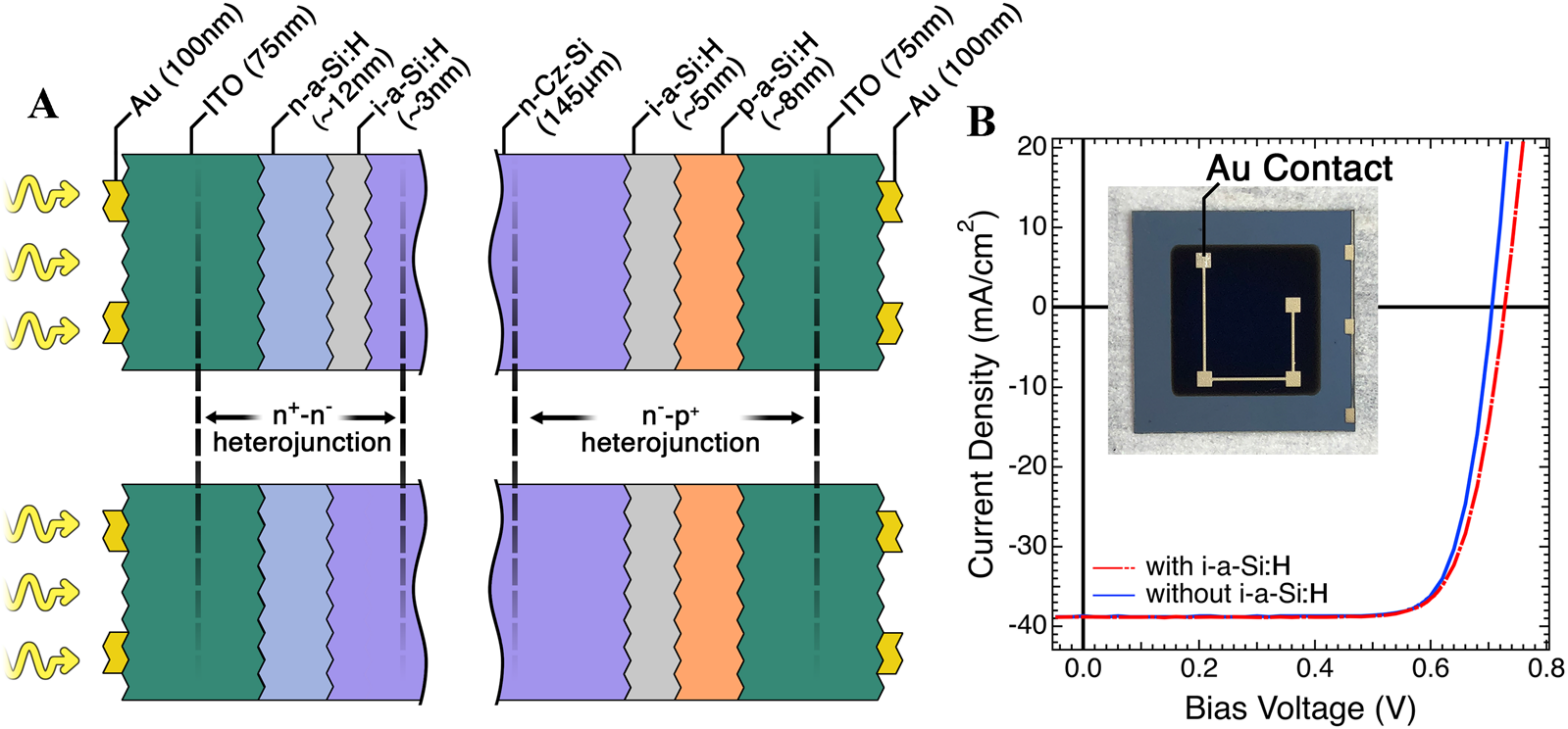
(**A**) Device structure diagram of the SC1-type (top) and SC2-type (bottom) SHJ SCs studied in this work including layer thicknesses. In the SC2 device, the 3 nm-thick intrinsic amorphous layer at the “front side” is omitted. The yellow arrows indicate the direction of illumination. (**B**) J(V) data of representative samples from original wafers under 1 sun illumination; dashed red line represents device with both a-Si layers. The inset shows an image of the device under test; the dark blue active area is ~ 1cm^2^.

In our experiments, we have studied solar cells with two slightly different structures, which we label SC1 and SC2 and are shown in Fig. 1A. These devices are of great interest for use in tandem perovskite/silicon solar cells in an effort to surpass the single-cell efficiency limit^3^. Importantly, the SHJ SC in the tandem devices are illuminated from the n-a-Si side, indicated in Fig. 1A, as the perovskite would be attached to the n-a-Si layer; we will refer to this side as the front side of the SHJ SC. The bulk of the SHJ SC consists of a 145 μm-thick, n-doped (~ 10^15^ dopants/cm^3^), single crystal silicon wafer prepared using the Czochralski method (*n-*c-Si). This wafer is patterned with a 3-dimensional pyramidal texturing for increased light in-coupling by reducing external reflection^27^. Grown on either side of this bulk is a thin layer of hydrogenated intrinsic amorphous silicon (*i-a*-Si:H) that serves as passivating layer which enhances V_OC_.

At the front side of the device, a 12 nm thick layer of *n*-doped hydrogenated amorphous silicon (n*-a*-Si:H) is added forming an n^+^-n^−^ heterojunction that serves to increase the selectivity for majority carriers^28^. An n^–^p^+^ heterojunction is formed at the back of the device, as shown in Fig. 1A, with the addition of an ~ 8 nm thick layer of *p-a*-Si:H, which serves as the p selective contact. This layer is doped to a degree that it can efficiently conduct hole currents towards the back contact without incurring losses; in the devices studied, the conductance is about 10^–3^ S/cm. However, the sheet resistance of the doped a-Si:H layer is too high for lateral current collection and hence SHJ SCs need an additional transparent conducting oxide layer, a 75 nm ITO layer in our case which also serves as anti-reflector, in combination with the 100 nm-thick gold contact as back mirror as shown in the inset of Fig. 1B. The structure of SC2 solar cell is in all regards identical to the SC1 except that the *i-a*-Si:H layer between *n-*c-Si and *n-a*-Si:H has been removed. As shown in Fig. 1B, this removal results, as expected, in a roughly 30 mV decrease in V_OC_.

Measurements of noise were carried out using a custom-built, low-temperature flow cryostat at temperatures ranging from 100 to 300 K. The samples were illuminated using narrow-spectrum red and yellow LEDs with wavelengths $\lambda = 625$ nm and $\lambda = 590$ nm, respectively, via a quartz-windowed vacuum port; all sources had a full width of 10 nm at half-maximum. A few noise spectra were obtained using a blue LED, $\lambda = 457$ nm. There was, however, no observable difference between these spectra and those taken with yellow light. Because of this, these spectra were not studied in a systematic way.

All noise measurement and illumination systems were mounted on an electrically isolated optical breadboard equipped with a pneumatic vibration isolation system and were powered using lead-acid batteries to mitigate noise injection. Importantly, relatively low photocurrent values were explored to avoid overwhelming the front-end transimpedance amplifiers.

The front-end cross-correlation circuit was constructed using commercial low-noise operational amplifiers placed inside the cryostat in very close proximity to the sample (within 3 cm), and thus operating at temperatures down to 77 K. Supplementary Figures S1 and S2 shows the principal circuit and physical implementation of the apparatus. Each transimpedance branch of this circuit amplifies the sum of the device noise and the amplifier’s own noise; these two signals are then cross correlated, as detailed in Refs.^24,29^. Due to the fact that noise signals of the individual amplifiers are uncorrelated, averaging the output over time allows for the effective removal of system noise, ideally leaving only the correlated noise signal from the device under test and greatly increasing the sensitivity of the measurement. In reality, the removal of the system noise is not complete and presents a noise floor of the form

1 $$S_{{floor}} = 2e_{n}^{2} \left[ {\frac{1}{{R_{D} }}\left( {\frac{1}{{R_{D} }} + \frac{1}{{R_{f} }}} \right) + \omega ^{2} C_{D} \left( {C_{D} + C_{i} + C_{{stray}} } \right)} \right],$$

where $e_{n}$ is the input voltage noise of the front operational amplifiers,$~R_{D}$ and $C_{D} ~$ are the resistance and capacitance of the device under test respectively,$~R_{F}$ is the feedback resistance of the transimpedance amplifiers, $\omega$ is the angular frequency, $C_{i}$ is the input capacitance of each channel, and $C_{{stray}}$ is the stray capacitance of the system. The very short distance between the amplifiers and the sample greatly reduces $C_{{stray}} ~$ and thus the overall sensitivity to external noise sources. Using this system, we have been able to probe signals 4 orders of magnitude below the noise floor of the front amplifiers. In Fig S3 of the supplemental material, we show spectra characterizing the apparatus performance using simple surface-mount resistors.

In all of our noise measurements, we were forced to restrict ourselves to the short-circuit configuration ($V = 0$) with varying light intensities due to the device capacitance. In principle, capacitance of multilayered devices can be a rather complex function of voltage^30^; it can also display useful correlations with noise (for example, in OLEDs we found one-to-one correlation between negative capacitance and generation-recombination noise term^31,32^). Unfortunately, our impedance measurements, shown in supplementary figure S4, could resolve only a single contribution due to the capacitance of the *n*^*–*^*p*^+^ heterojunction, which displayed the expected divergent behavior $1/C^{2} \propto \left( {V_{{OC}} - V} \right)$ observed in previous works^33,34^. Close to $~V_{{OC}}$, divergent capacitance produces a very large $f^{2}$-term in the noise spectra which obscures the data of interest.

When taking the measurements, the system was illuminated for roughly 30 min to allow a steady state condition to form. The noise signals were then recorded and averaged over 3000 iterations. To account for the effect of the residual noise floor, an initial data series was taken in the dark with 50,000 iterations. This was then subtracted from the raw data obtained at different light intensities; the resulting noise spectra are those shown and analyzed in the rest of the paper.

## Experimental results

**Figure 2:**
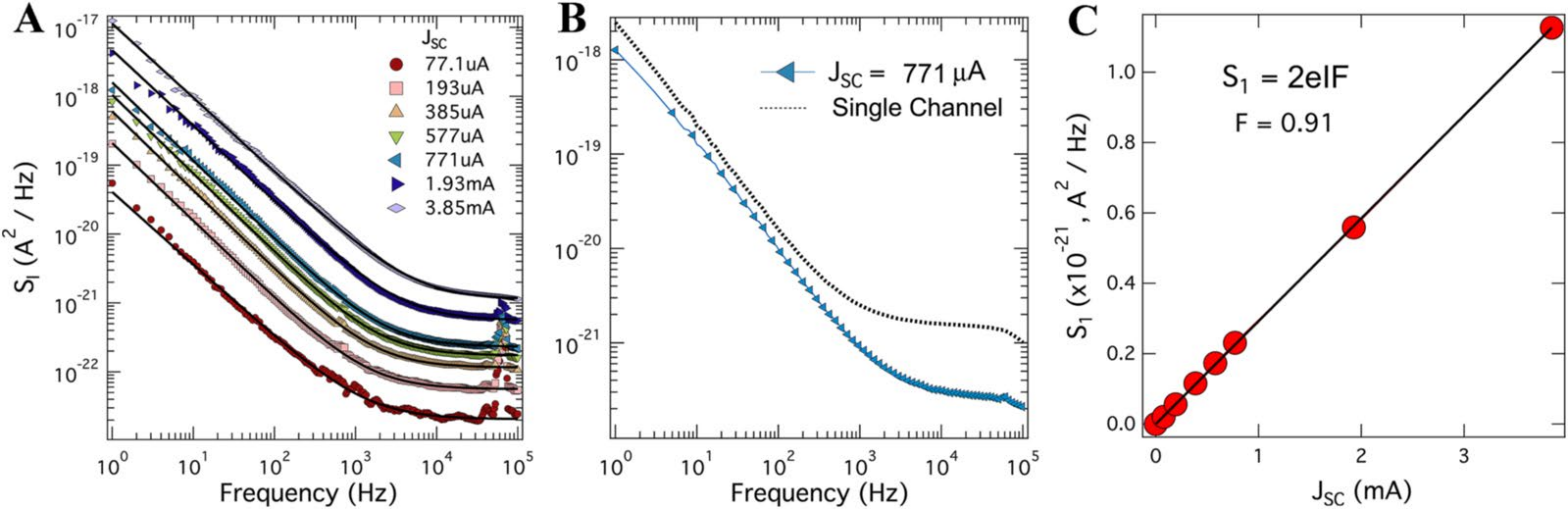
Current spectral density measured with the cross-correlation technique of the SHJ solar cell SC1 at short-circuit condition under red light illumination. (**A**) Frequency dependence of current noise power spectral density at 300 K under illumination with varying intensity from a narrow-spectrum red LED ($\lambda = 625\;{\text{nm}}$). The legend indicates short-circuit DC current in the device. Black solid lines are fits to Eq. (1). (**B**) Comparison of a cross-correlated data set to that of a single channel showing the dominance of the front-amplifier noise on the single channel thus necessitating cross correlation. (**C**) The magnitude of frequency-independent term extracted from fitting to Eq. (2) versus short-circuit current. Solid line is a linear fit to the data, corresponding to a Fano factor $F \approx 0.9$.

In Fig. 2A, we show the current noise power spectra versus frequency for the SC1 device under illumination by the red LED source ($\lambda = 625\;{\text{nm}}$) at room temperature; the legend indicates the short-circuit current. Two clear features emerge from these spectra. First is a strong $1/f$ term extending to approximately 1 kHz. The second feature is the clear plateau of a frequency-independent term. The high-frequency features observed in the plateaus around 50 kHz are artifacts from an external source, likely injected into the system due to a ground loop.

To analyze the data quantitatively, we fit each curve to following equation,

2 $$S_{I} = S_{1} + \frac{{S_{2} }}{{f^{a} }}$$

Here, the first term represents a frequency-independent term comprised of shot noise and thermal noise. The second term models a $1/f$-like flicker noise. Figure 2B illustrates the importance of the cross-correlation method in detecting the true frequency-independent plateau. The dashed line shows the noise taken with the single-channel input; that is what one typically gets with a standard spectrum analyzer (for comparison also see Fig. S3 in the supplementary material). This signal is dominated by the noise of the front-end amplifier which is typically orders of magnitude greater than the signal of interest.

From the fits, we find the exponent of the flicker component to $a \approx 1$ across all illumination intensities. In Fig. 2C, we present the magnitude of the frequency-independent term, $S_{1}$, as a function of the short-circuit current, $J_{{SC}}$. This dependence is linear, giving strong evidence that this term is completely dominated by the shot noise present in the device; at the measured photocurrents, we find the expected thermal noise ceiling to be several orders of magnitude smaller that the shot noise term. The fit to the equation $S_{1} = 2eIF$, shown as a solid black line in Fig. 2C, returns a reasonable value of the Fano factor $F \approx 0.9$. As we argue below, this indicates that the shot noise term represents a single process, most likely holes traversing the n^–^p^+^ heterojunction. It is important to note that the correct value of shot noise can only be obtained using the cross-correlation method. In measurements using a standard spectrum analyzer, represented in our case by a single channel output, the dashed line in Fig. 2B, the frequency-independent term is heavily dominated by the noise from the front amplifier.

**Figure 3:**
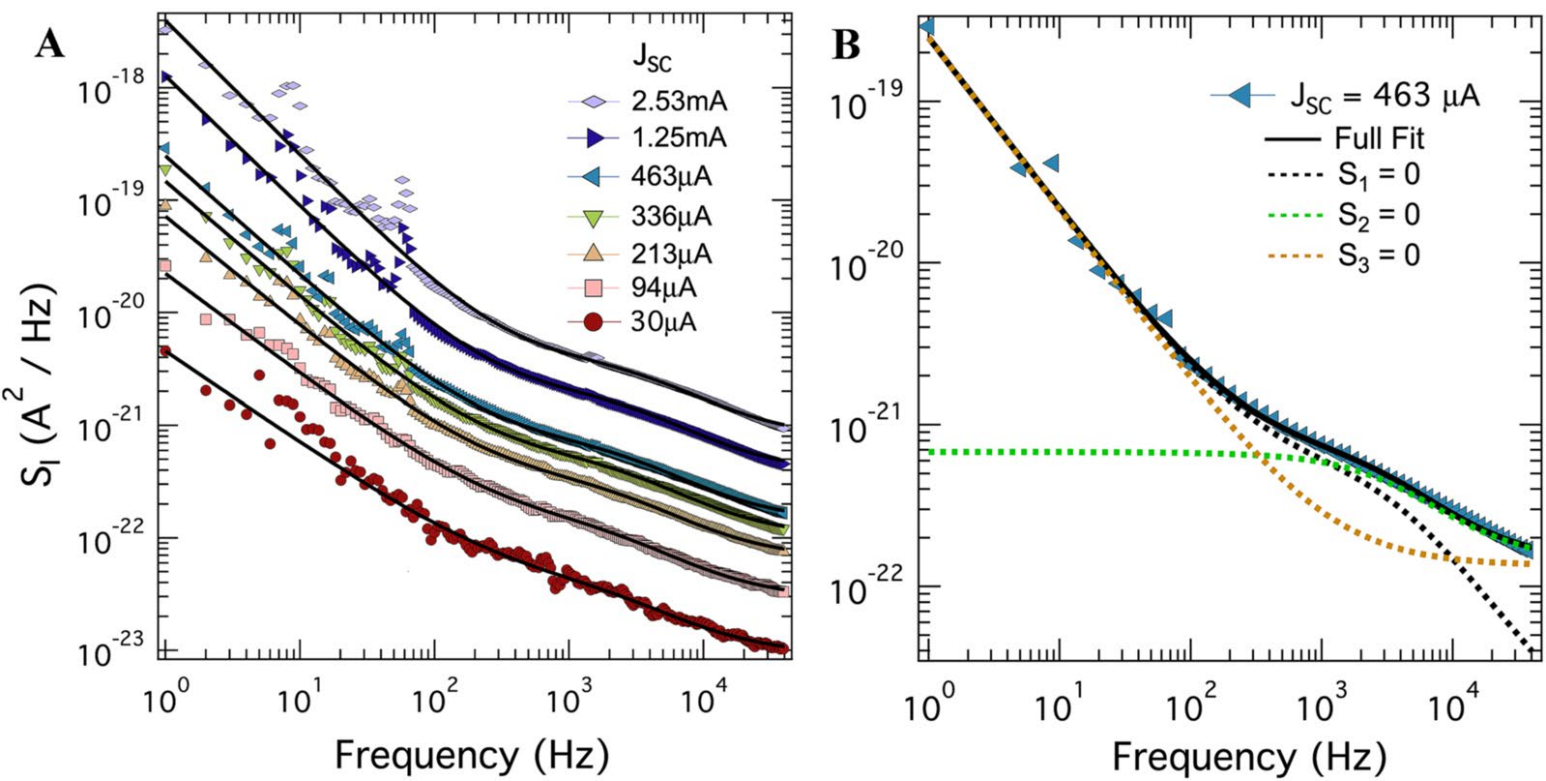
Current spectral density of the SHJ solar cell SC1 in short-circuit configuration under yellow light. (**A**) Frequency dependence of current noise power spectral density at 300 K under illumination with varying intensity from a narrow-spectrum yellow LED ($\lambda = 590~\;{\text{nm}}$). The legend indicates short-circuit DC current in the device. Black solid lines are fits to Eq. (3). (**B**) A representative noise spectrum corresponding to *J*_SC_ = 463 μA, where each term in Eq. (3) is sequentially set to zero to illustrate the presence of both frequency-independent and generation-recombination noise.

The noise power spectral densities of the same device, this time illuminated by a yellow LED source ($\lambda = 590\;{\text{nm}}$), are shown in Fig. 3A. Compared to red light, a new feature clearly emerges between 1 and 10 kHz which we will refer to as GR1. To account for this feature, we must introduce a third term to the fitting equation,

3 $$S_{I} = S_{1} + \frac{{S_{2} }}{{f^{a} }} + Re\left[ {\frac{{S_{3} }}{{1 + \left( {i\omega \tau } \right)^{{1 - b}} }}} \right]$$

This term represents a generalization of generation-recombination noise, allowing for the dispersion of recombination times, $\tau$, as determined by the exponent *b*. Note that when *b* = 0, this term takes on the familiar form of a Lorentzian profile. To illustrate that this feature is not merely an artifact, Fig. 3B shows a representative data set for a single illumination with multiple fits to Eq. (3), each with a different term set to zero. The orange dashed line represents a fit identical to those in Fig. 2A in which $S_{3} = 0$ making it obvious this feature was not present under red light. As is apparent, all three terms are needed to fit the data.

**Figure 4:**
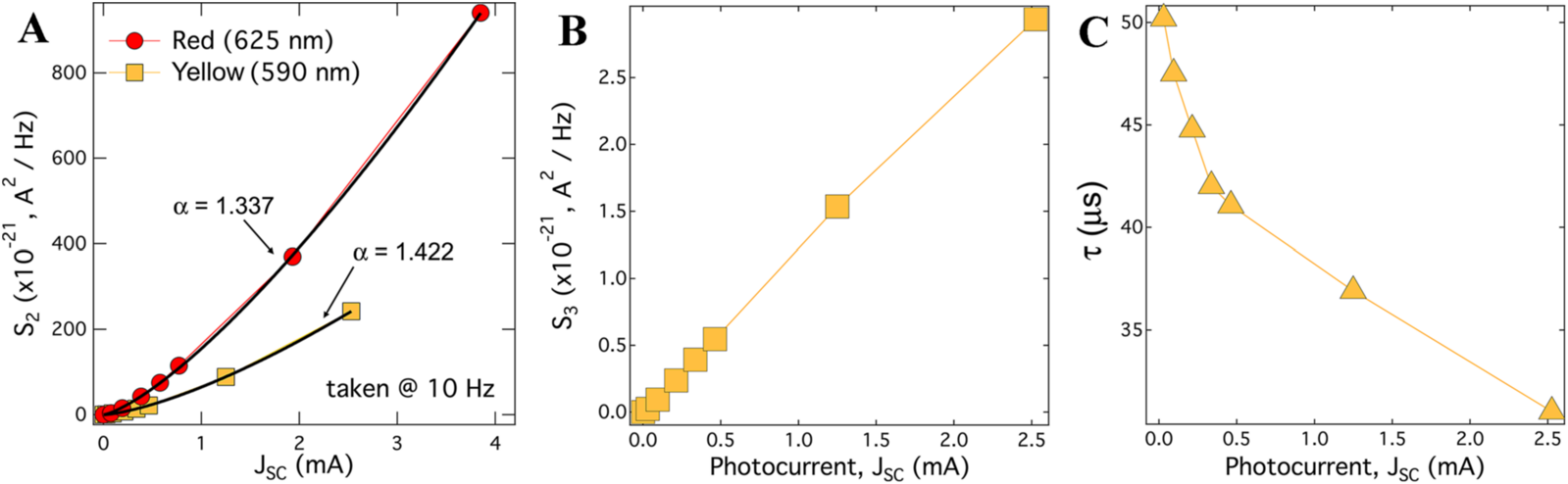
Extracted parameters from current noise spectra shown in Fig. 3. (**A**) The magnitude of the low frequency $1/f$ noise at 10 Hz as a function of short-circuit current for illumination with red and yellow light. The black curves represent fits to a power law, $S_{2} = A \times J_{{SC}}^{a}$. (**B**) The magnitude of the generation-recombination term GR1 extracted from a fit to Eq. (3) as a function of *J*_*SC*_; the value of the exponent of the generation-recombination term is *b* = 0.21. (**C**) The GR1 time constant plotted as a function of *J*_*SC*_.

We note that the frequency-independent term is also critical in finding a good fit to Eq. (3), as shown by the black dashed line in Fig. 3B. In this curve, $S_{1} = 0$. It was difficult, however, for the curve fitting algorithm to capture the value of $S_{1}$ as a fit parameter. To account for this, we assumed a shot noise with the same Fano factor that was observed under red light, $F = 0.9$. Thus, the value of $S_{1}$ was fixed for each photocurrent measured, independent of illumination wavelength. We also point out that the value of the dispersion exponent *b* was determined using the lowest photocurrent spectrum and was then fixed for the remaining fits. The parameters extracted from these fits are presented in Fig. 4.

To clarify further the origin of the different noise terms, we performed the same series of tests on the SC2 device in which the passivating *i-a-*Si:H layer between the bulk *n-*c-Si and *n-a-*Si:H layer was omitted. In fact, this device was essential to our initial motivation to understand if noise spectroscopy can characterize trap states on the heterointerface of c-Si and the ability of *i-a-*Si:H to passivate it. To our surprise, no quantitative difference was detected between the spectra generated by the SC1 and SC2 devices as shown in Fig. S5 of the supplementary material. Despite the obvious effect on $V_{{OC}}$, the absence of the front-facing *i-a*-Si:H layer does not appear to produce an observable effect upon the noise spectra indicating that the noise features seen do not relate to the passivation quality or to the transport properties in the *i*-a-Si:H layer.

**Figure 5:**
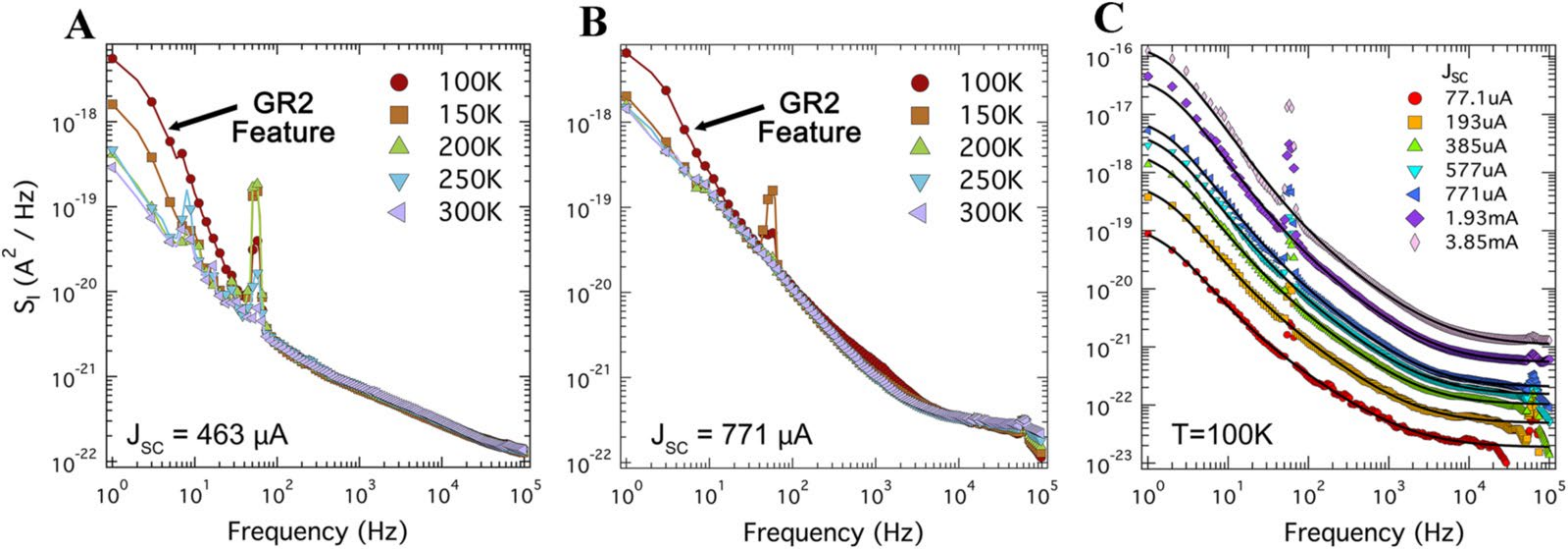
Current spectral density as a function of temperature of the SHJ solar cell SC1 at short-circuit condition. (**A**) The frequency and temperature dependence of the current noise spectra under yellow light illumination, illustrating the near temperature-independence of the GR1 noise term (frequency range 100 Hz–10 kHz). This figure also illustrates an emerging low-temperature Lorentzian feature (GR2 term, $\tau \approx 0.1\;{\text{s}}$) with decreasing temperature. The noise peak occurring at ~ 60 Hz is caused by mechanical noise introduced by the flow of liquid nitrogen. (**B**) The frequency and temperature dependence of the current noise spectra under red light. Again, the GR2 spectrum with $\tau \approx 0.1\;{\text{s}}$ is detected below 100 K. (**C**) The full set of spectra under red light at 100 K. The black curves represent a fit to the sum of Eq. (4).

In our final experimental test, we performed noise measurements on the SC1 device at temperatures down to 100 K, the results of which are shown in Fig. 5. The only noticeable change in spectra is the appearance of a new pronounced generation-recombination term at low frequencies and temperatures below about 100 K, indicated in the figure with an arrow. We will refer to this feature as GR2.

We found that this GR2 term can be approximated by a simple Lorentzian, $S_{4} /\left[ {1 + \left( {f t } \right)^{2} } \right]$, with roughly the same relaxation constant for both red light ($t \approx 0.12\;{\text{s}}$) and yellow light ($t \approx 0.1\;{\text{s}}$). This term was then added to the fitting equation, now expressed as

4 $$S_{I} = S_{1} + \frac{{S_{2} }}{{f^{a} }} + Re\left[ {\frac{{S_{3} }}{{1 + \left( {i\omega \tau } \right)^{{1 - b}} }}} \right] + \frac{{S_{4} }}{{\left[ {1 + \left( {ft} \right)^{2} } \right]}}$$

**Figure 6:**
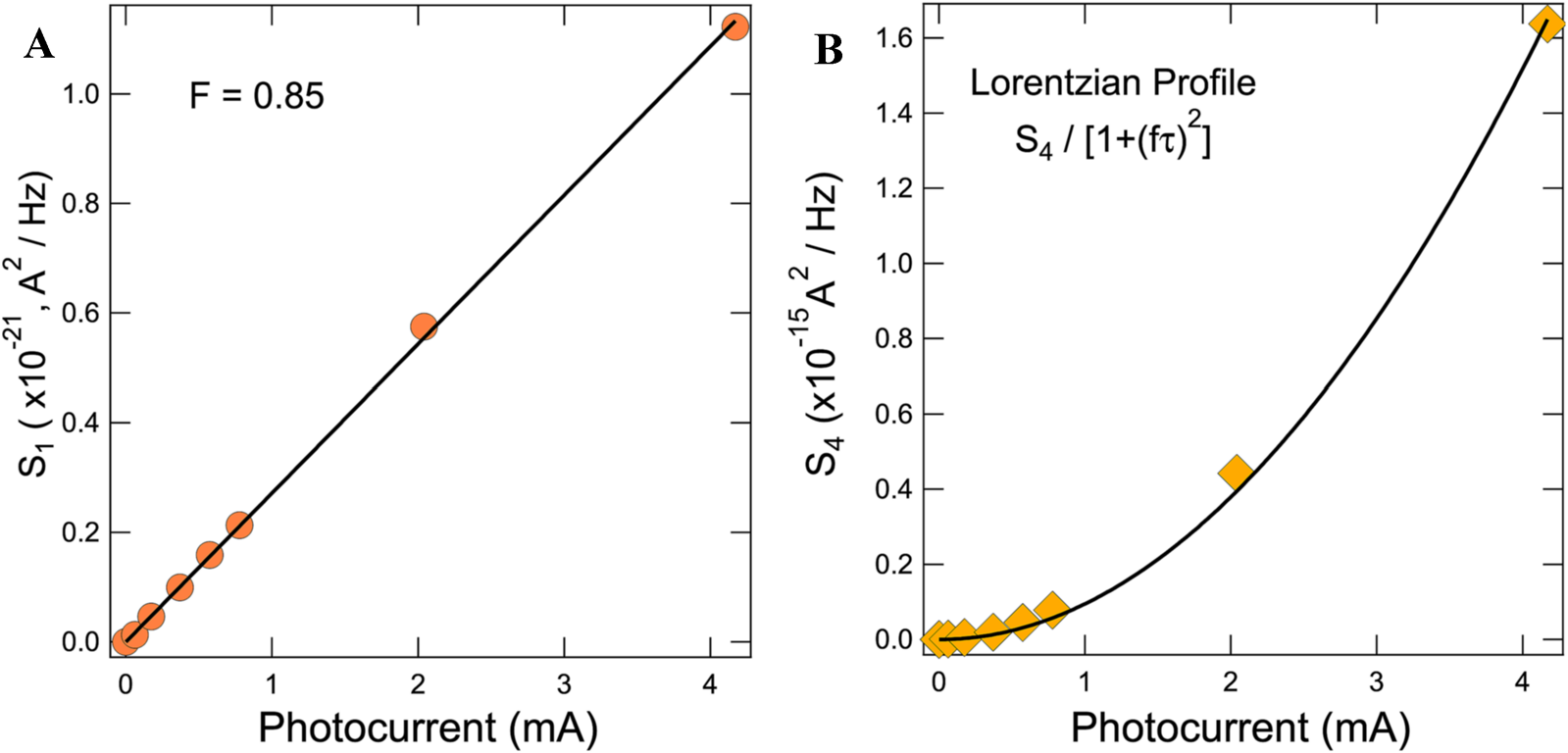
Extracted parameters from current noise spectra taken at T = 100 K and shown in Fig. 5. (**A**) The magnitude of the frequency-independent term extracted from the fits to Eq. (4) in Fig. 5C as a function of photocurrent. The minimal reduction of the Fano factor as compared to the room-temperature data presented in Fig. 2 illustrates that this term is true shot noise. (**B**) The magnitude of the low-frequency Lorentzian term in Eq. (4) seen to emerge below 150 K as a function of photocurrent. The black line indicates a fit to an $J_{{sc}} ^{2}$ dependence.

The magnitude of the shot noise and GR2 terms that result from the fits to Eq. (4) of the low-temperature spectra are shown in Fig. 6A,B, respectively. We note that the frequency-independent term has changed very little from the room temperature measurements, illustrating that this term is indeed completely dominated by shot noise. We find that the magnitude of the GR2 term closely follows a $J_{{sc}} ^{2}$ dependance.

## Discussion

**Figure 7:**
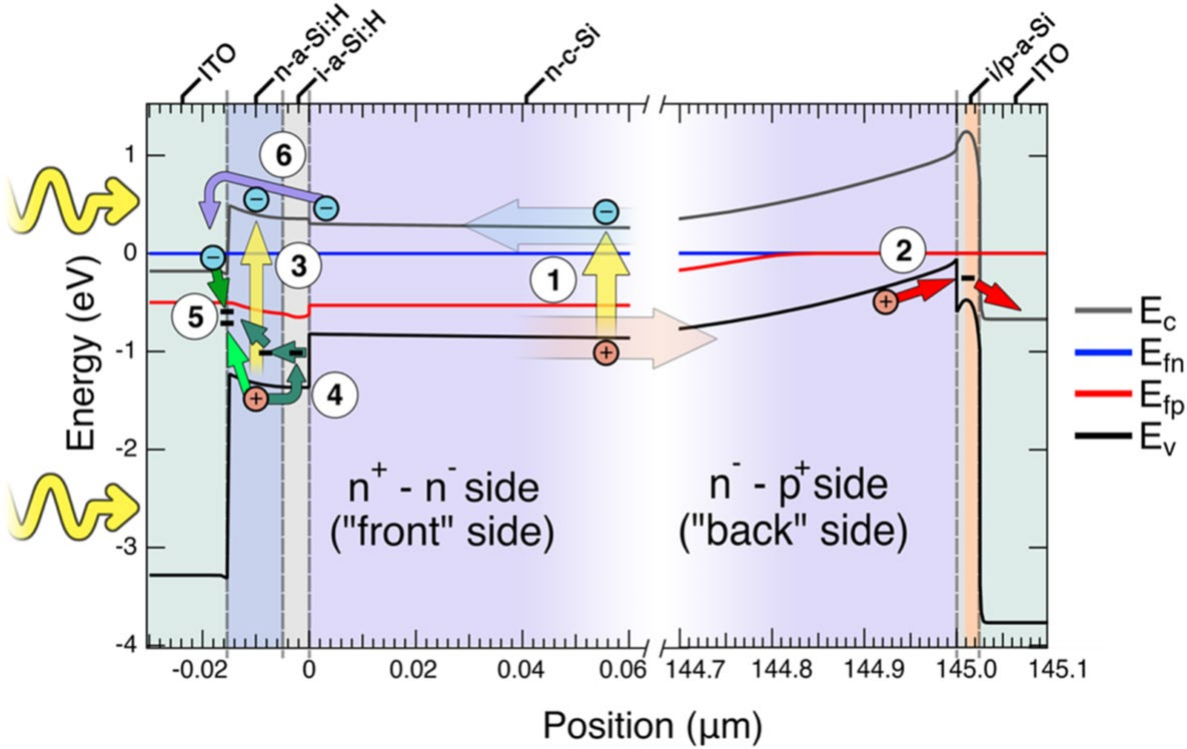
Band diagram sketch from TCAD-SENTAURUS simulation of the complete device SC1 under yellow light illumination corresponding to a current density of 2 mA/cm^2^; the n and p quasi-Fermi levels are shown as blue and red lines, respectively. The band bending at the front side is not noticeable on the scale presented. Circled numbers highlight the noise processes contributing to the noise spectra: (1) photogenerated carriers in the bulk, (2) trap-assisted tunneling through the *ip*^+^-a-Si:H, and (3) photogenerated carriers in the *in*^+^-a-Si:H region, (4) trapping of holes in defects in the *in*^+^-a-Si:H and (5) recombination with electrons at the ITO/ *in*^+^-a-Si:H interface, (6) thermally assisted passage of electrons over the *in*^+^-a-Si:H barrier.

Let us now discuss the individual contributions to the noise spectra of SHJ SCs as described in Eq. (4) and the possible microscopic processes generating them. This analysis was assisted by computer simulation using the 1D and 2D numerical simulation package TCAD-SENTAURUS that allows for the inclusion of trap-assisted and direct tunneling at the relevant interfaces^35^; Fig. 7 shows a sketch of the resulting band diagram of the SC1 device under yellow light illumination for the case of *J*_*SC*_ = 2 mA/cm^2^, which is in the range of illumination levels used in the experiment. Importantly, trap-assisted tunneling was enabled on the front and direct tunneling on the back side, though it turned out not to play an important role for the band profile; in most simulations, these processes are ignored due to the small effect on photocurrent. The vast majority of the charge carriers are generated far from the SHJ interface in the bulk (process 1) but photogeneration in the thin *a*-Si:H layer at the front side (process 4) was also taken into account in the band calculation shown in Fig. 7. This is shown in more detail in Figs. S7 and S8 in the supplementary material.

## Frequency-independent term, S_1_

To first approximation, the total noise in the device can be represented by a series of noise sources, $i_{n}$, each self-shorted by its own internal resistance,$~R_{n}$. Here the sub-index *n* indicates distinct elements of the stack and the interfaces between them. From Kirchhoff’s law, the total noise current seen at the contacts is given as

5 $$I_{T} = \mathop \sum \limits_{n} \frac{{i_{n} R_{n} }}{{R_{T} }}$$

where $R_{T}$ is the total device resistance. To be more specific, let us consider a series collection of sources $,~i_{n}$, which represent uncorrelated shot noise with a full scale Schottky value $S_{n} = 2eI$. Thus, the total current noise seen at the electrodes is

6 $$S_{T} = I_{T}^{2} = \mathop \sum \limits_{n} i_{n}^{2} \left( {\frac{{R_{n} }}{{R_{T} }}} \right)^{2} = 2eI\mathop \sum \limits_{n} \left( {\frac{{R_{n} }}{{R_{T} }}} \right)^{2} = 2eIF,$$

where the Fano factor, *F*, is determined by the resistor network and characterizes a suppression of the overall shot noise. For example, for *N* identical sources connected in series, the expected Fano factor would be *F* = *1/N*^36^. We have verified the efficacy of this approach by performing noise measurements on identical forward-biased diodes, arranged in 1-, 2-, and 3-diode series configurations. As expected, the Fano factor scaled with the number of diodes, yielding *F* = 0.48 for two diodes and *F* = 0.35 for three.

The S_1_ term can unambiguously be assigned to shot noise as it follows the expected form $S_{1} = 2eIF$ with almost the same value of Fano factor at room temperature, *F* = 0.9, and at 100 K, *F* = 0.85 as shown in Fig. 6A. The lack of temperature dependence rules out thermal noise as an alternative mechanism behind this term. The fact that the Fano factor is observed to be so near unity indicates that the shot noise is dominated by contributions from a single source within the device. Referring to Eq. (6), the presence of additional shot noise sources, either individual interfaces or hops within a single interface, would greatly reduce the Fano factor. This illustrates the spatial selectivity property of the CCNS method and its ability to isolate the effect of a single interface.

In the SC1 SHJ SC, there are potentially three processes producing shot noise: tunneling or thermally activated transition of minority carriers across the *n-a*-Si:H / *i-a*-Si:H / *n-*c-Si heterojunction near the front side of the device (process 6 in Fig. 7), light-induced electron–hole pair generation either in the *n-*c-Si bulk (process 1) and in the *ni*-a-Si:H layer (process 4), and tunneling of majority carriers across the *n-*c-Si / *i-a*-Si:H / *p-a*-Si:H junction and into the electrode at the back side of the device (process 2 in Fig. 7). The last of these sources has by far the highest resistance; the *i*-a-Si:H layer on the back side is thicker than on the front (see Fig. 1A) and the *n*^*–*^*p*^+^ heterojunction is wider and more depleted than the front-facing n^+^-n^-^ heterojunction. Therefore, by Eqs. (5) and (6), the total measured shot noise of the SHJ SC must be dominated by process 2 in Fig. 7.

Focusing on this single noise element and using the same arguments as above, we notice that the near-unity value of the Fano factor strongly suggests that the transition across the n^–^p^+^ heterojunction occurs either in a single step or in a sequential multi-step propagation (not included in the simulation) with one rate-limiting transition. In the language of Eqs. (5) and (6), this limiting transition has the highest “bottle-neck” resistance, although details of the microscopic process cannot be resolved using this technique alone.

## Flicker 1/f-like term, S_2_

Equations 5 and 6 describe a standard procedure for analyzing noise in electronic circuits composed of lumped elements. It has been successfully extended to analyze noise generated by hopping transport in inorganic^37^ and organic semiconductors^25,26^, where the high value of the Fano factor was taken as an indication of a small number of “hard” hops with a very high resistance. More recently, we have used this approach to analyze the transport processes which occur in a modern methylammonium lead triiodide perovskite solar cell^38^.

Importantly, we note that the concept that the contribution of a noise source in the most resistive element is greatly amplified is general and can be used when discussing *any* noise source. However, the extension to 1/*f* is not as straightforward as for shot noise given that there is no fundamental mechanism fixing the magnitude for those noise sources, making it difficult to write an expression for $i_{n}^{2}$. In complex, multi-layer devices, the resistive elements could represent an interface, and individual layers, or a group of layers such as a p–n junction.

We anticipate this noise component is again dominated by a process in the element of the SHJ SC which has the highest resistance, the n^–^p^+^ heterojunction. Indirectly, this assertion is supported by the fact that the same magnitude of $1/f$ noise is observed in both the SC1 and SC2 devices. The n^–^p^+^ heterojunction collects holes created in *n*–c-Si wafer upon photoexcitation. The holes encounter a potential barrier with a height $\Delta E_{b} \approx 0.4\;{\text{eV}}$ at the interface between *n-*c-Si and *i-a*-Si:H, shown as process 2 in Fig. 7. This barrier forms as a result of the thin depletion zone within the *ip*-Si:H region.

The observed deviation in the dependence of the power spectral density on current, $S_{2} = AJ_{{SC}}^{\alpha }$, (see Fig. 4A) from its standard value $\alpha \approx 2$ implies that the noise comes not from the existing defects but rather from ones induced by the current^39^. Most likely, the 1/*f* noise originates from the modulation of the local height of potential barriers and, in turn, the local probability of tunneling, induced by the trapping and release of charges at the interfaces. This is supported by the fact that S_2_ is temperature independent. To avoid confusion, we note that in our model, process 2 contributes to both shot noise and 1/*f* noise: each conduction channel contributes to shot noise due to the discreteness of the charge, but it is the fluctuation in the conduction of _these_ channels over time that is responsible for 1/f noise. This assertation is consistent with the recent observation of trap-assisted tunneling in conjunction with random telegraph noise by conductive atomic force microscopy in the a-Si:H^40^. However, we cannot completely reject a possibility that at least part of the 1/*f* noise comes from current-induced migration of hydrogen atoms as such a process could cause local fluctuations in device conductance as the percolation path of carriers is modulated by slight shifts in crystal structure^41^.

## GR1 generation-recombination term, S_3_

This term, shown in Fig. 5A,B, has several peculiar properties. The magnitude does not follow the $J^{2}$ dependance (Fig. 4B) expected for systems where charge carriers probe existing traps of defect states. Its relaxation time, 30–50 μs (Fig. 4C), only weakly decreases (by a factor of two-fold) when electrical current changes by two orders of magnitude. Remarkably, both the magnitude and relaxation time are temperature independent. Furthermore, the GR1 term is absent under illumination by red light.

From our simulation, we see that all light is absorbed within the first 10–20 μm of the device as shown in Fig. S6 of the supplementary material. Because the GR1 term depends on the wavelength of the light, it must originate in the elements of the device where light is absorbed. Therefore, it cannot come from the back-facing *n*^*–*^*p*^+^ heterojunction and must be localized to the front-facing *ni*-a-Si:H contact.

We suggest that this term is linked to a multi-step relaxation of holes in the a-Si:H layer leading to a loss current induced by the space charge region at the ITO/p-a-Si:H interface (Fig. 7). This process begins with the photogeneration of an electron–hole pair within the a-Si:H shown as process 3 in Fig. 7. The hole then relaxes into a defect state located at the ITO/n^+^-a-Si:H interface either directly or via an intermediate bandtail or defect state within the ITO (process 5). That this process is temperature-independent can be explained by the fact that it is a purely quantum mechanical relaxation process; the hole simply loses energy and doesn’t need to overcome any energy barrier.

The wavelength-dependence is more challenging to interpret and the simulation turned out to be crucial to our understanding of the effect. It shows that when changing from red to yellow light, the hole concentration in the *in*-a-Si:H region increased by a factor of 3, as shown in the supplementary figure S7. A three-fold decrease in magnitude of GR1 shifts it below the noise contributions present in the spectrum, rendering it undetectable under red light as observed. For example, at 2 mA/cm^2^, the magnitude of GR1 is $S_{3} = 3\;{\text{A}}^{2} /{\text{Hz}}$ (see Fig. 4B). Decreasing this by a factor of three reduces it below the value of shot noise, $S_{1} = 1.2\;{\text{A}}^{2} /{\text{Hz}}$, at this same photocurrent (see Fig. 2C).

Overall, the effect of these recombination events within at the ITO/ *in*-a-Si:H on the device current is small when compared to the sheer number of photogenerated charge carriers. Indeed, these effects are typically ignored in device simulations to simplify the process. The noise they generate, however, is substantial enough to be detected and show that this CCNS method is sensitive enough to isolate and study these small recombination effects as a means to understand interfacial processes occurring within the amorphous layers. For example, it is known that high-temperature annealing helps improve device performances due to some kind of change at the ITO/a-Si boundary^42^, though the nature of this change is not well understood. We suggest that the CCNS method could be used to explore the generation of defects at this boundary and improve solar cell performance so close to the theoretical maximum.

## GR2 generation-recombination term, S_4_

This term has a single relaxation time with zero dispersion, meaning it is created by a mono-energetic level. Further, it has an $I^{2}$ dependence, meaning that it probes an existing level/trap in the device. It is not present at room temperature and emerges at about 100 K. The characteristic timescale of the processes can be estimated as $t \approx 1/\left[ {\upsilon _{0} \times {\text{exp}}\left( { - \frac{{\Delta E}}{{k_{b} T}}} \right)} \right]$, where $\upsilon _{0} \sim10^{{12}} \;{\text{s}}^{{ - 1}}$ is the typical attempt frequency assumed for a-Si:H^43^. The appearance of the GR2 term with $t \approx 100$ ms at temperature about 100 K places the estimate for the energy level at $\Delta E$ = 0.2–0.25 eV. Due to the complexity of the band diagram and the large number of potential noise-generating processes present in the device, identifying the source of GR2 using noise measurements alone is difficult, but from Fig. 7 we see only one process that satisfies all three of the above criteria—the thermally-assisted passage of the electrons over the barrier in the *in*-a-Si:H, shown as process 6.

### Conclusions

In this paper, we have studied the noise properties of SHJ SCs in the temperature range 100–300 K and under illumination by monochromatic red (625 nm) and yellow (590 nm) light. These measurements were done in the short-circuit configuration using a cross-correlation method in a current-monitoring configuration. We observe the noise spectra to contain a rich set of information. First, we observe a near-unity Fano factor shot noise contribution, indicating the dominance of the holes tunneling through n^–^p^+^ heterojunction given the greater resistance and higher energy barrier in this region. Second, the $1/f$ contribution was found to be temperature-independent and related to photocurrent as $J^{\alpha }$. The observed exponent $~\alpha \approx 1.3 - 1.5$, instead of standard $\alpha = 2$, suggests that this contribution reflects not the existing defects but rather the presence of random telegraph noise that is induced by local potential fluctuation due to trapped charges at a-Si:H defects leading to the switching of tunneling transitions. Third, we observe a generation-recombination term (GR1) reflecting trap-assisted tunneling and recombination in the n-doped amorphous Si layers at the front side of the device. This signal disappears under red illumination as under these conditions, recombination is reduced and the noise signal falls below the shot noise floor. Finally, a second generation-recombination term (GR2) with a single relaxation time appears at temperatures below around 100 K appearing to correspond to the thermally assisted passage of electrons over the ITO/n-a-Si:H interface.

Summarizing, we have shown that current noise spectra are typically dominated by the contributions from the most resistive elements in the device stack. As in the studied SHJ SC the most resistive element is the depletion layer containing the *i-* and *p-*doped a-Si:H, we conclude that it is responsible for the observed shot and $1/f$ noise terms. However, since the monochromatic light used here does not generate e–h pairs anywhere near the back interface and GR1 term does display the sensitivity to the light wavelength, it must be attributed to the front interface. It also follows that GR2 must also be associated with the smaller energy barrier to electrons at the front interfaces as the back interface barrier becomes insurmountable by thermal processes at lower temperatures.

The complex planar stacks are widespread in modern solar cells and light emitting diodes, so the unappreciated space selectivity of current noise spectroscopy could potentially make it a useful and non-invasive complimentary characterization tool, particularly when implemented in a cross-correlation configuration.

## Supplementary Information

Supplementary Information.

## Data Availability

The datasets generated and analyzed in this work are available from the corresponding author upon reasonable request.
