# Supplementary material for: Relaxation processes in silicon heterojunction solar cells probed via noise spectroscopy
Source: Sci Rep. 2021 Jun 24;11:13238. doi: 10.1038/s41598-021-92866-w (PMC8225850; doi:10.1038/s41598-021-92866-w)
Supplement: Supplementary file 1 — Supplementary Information. [file 41598_2021_92866_MOESM1_ESM.docx]

Supplementary Material

**Relaxation processes in silicon heterojunction solar cells probed via noise spectroscopy**

Kevin Davenport^1*^, C. T. Trinh^2^, Mark Hayward^1^, Klaus Lips^1,3^, and Andrey Rogachev^1^


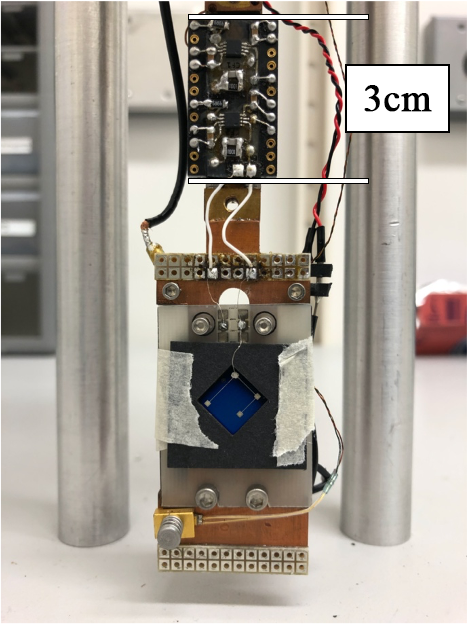


**Supplemental Figure S1.** A photograph of the SC1 device connected to the cross-correlation spectrum analyzer. The sample can be seen in the bottom-center with the active region (dark blue) being masked to avoid activating the surrounding wafer. Gold wire is connected to the electrodes on either side using silver paint. The close proximity of the low-noise front-end amplifiers (seen top-center) reduces with capacitance of the input lines, increasing the apparatus’s bandwidth.


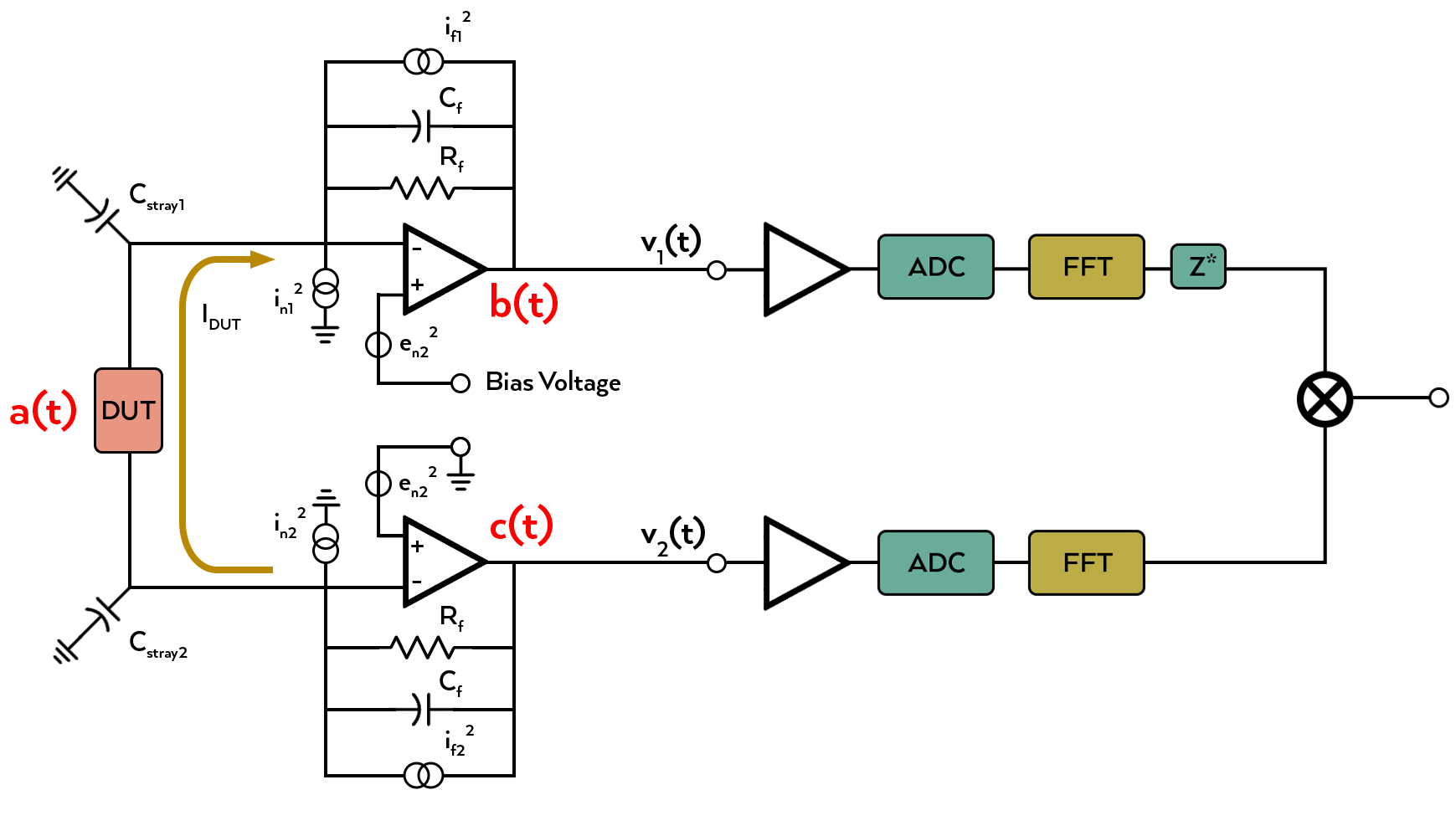


**Supplemental Figure S2.** A schematic of the front end of the current noise cross-correlating spectrum analyzer used in the experiment. The outputs of the two transimpedance amplifiers, v_1_(t) and v_2_(t), are a sum of the noise from the device under test and the single-channel amplifier noise. All measurements were taking at a bias voltage of 0 V to avoid capacitive effects. In the actual circuit, the operation amplifiers used were the low input noise TI OPA211. The values of the R_f_ and C_f_ in each channel identical and chosen to maximize the bandwidth of the device and stabilize the circuit; typical values of R_f_ were 1kΩ and 10kΩ and the typical value of C_f_ was 100pF. The individual channels were output to a National Instruments NI-6366 USB digital acquisition card and fed into custom LabView software which performed the Fourier transformation and cross-correlation to produce the spectra presented in the paper.


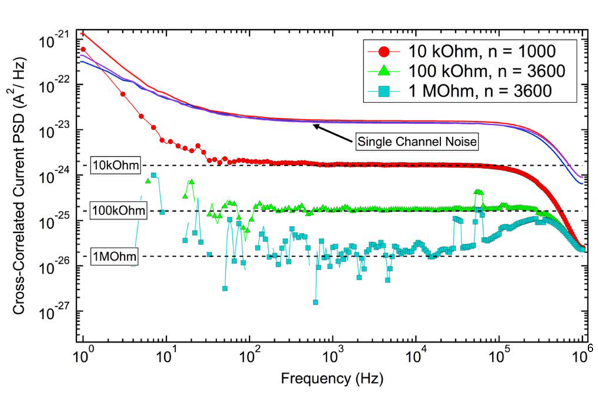


**Supplemental Figure S3.** The room temperature current noise spectra of three simple, surface-mount resistors using the apparatus shown in SF1 illustrating the efficacy of the cross-correlation method. The single-channel noise is shown to be nearly identical for all three. The black dashed lines represent the theoretical value of each resistor’s Johnson-Nyquist current noise, $S=4kTG$, where *G* is the conductance of each device.


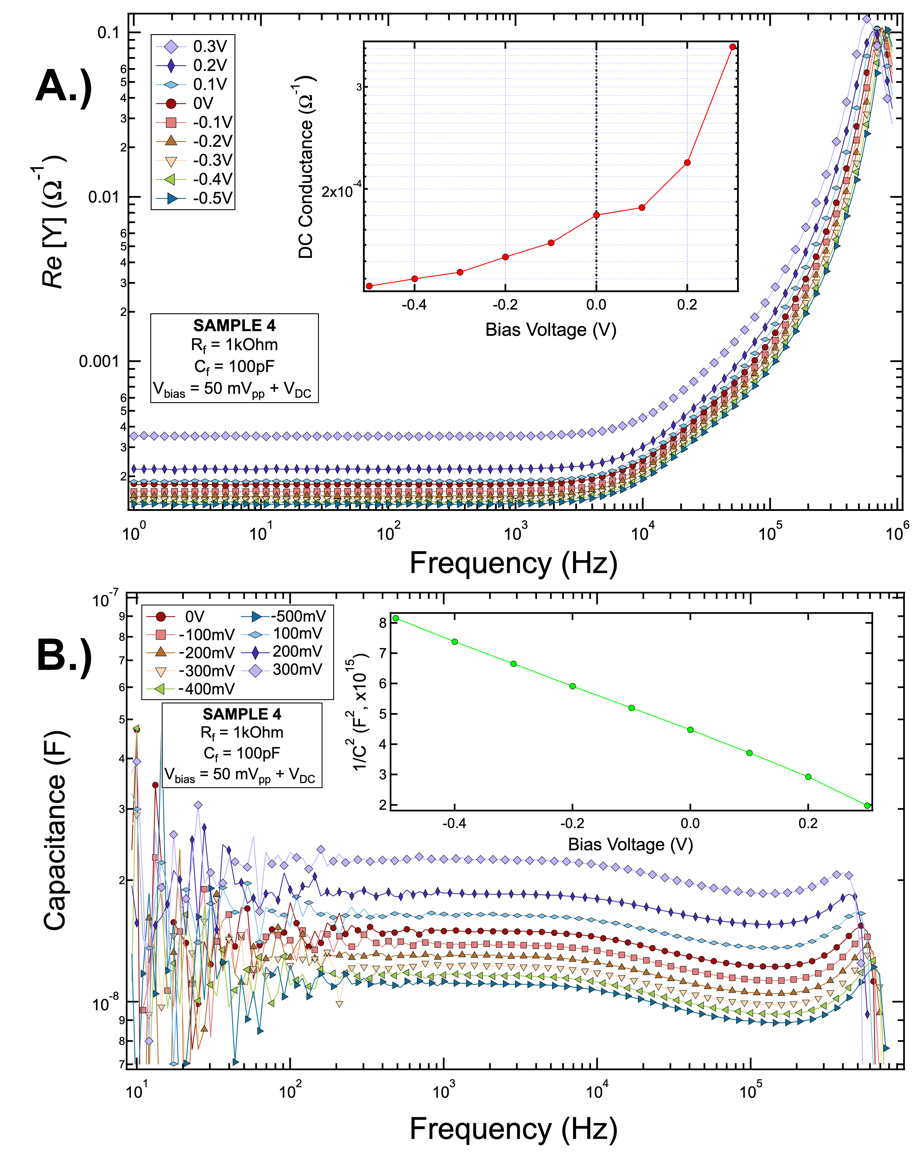


**Supplemental Figure S4.** Admittance spectroscopy results from the SC1 device. A.) Device conductance from the real part of admittance as a function of frequency at changing bias voltage. The values of the plateau regions are plotted in the inset as a function of bias voltage showing the expected increase in conductivity with increasing bias. B.) Capacitance from the imaginary part of admittance as a function of frequency at changing bias voltage. The value of capacitance was taken as the plateau value between 100Hz and 10kHz. The inset shows 1/C^2^ versus bias voltage showing a divergent value of capacitance as the bias approaches V_OC_. The slope of the inset yields a carrier concentration of ~7x10^15^ dopants/cm^3^.

**Supplemental Figure S5.** A comparison of the current noise spectra measured for the SC1 device (shown in open blue markers) which contains both front and back *i*-a-Si:H layers, and the SC2 device (shown in solid red markers) in which the front-facing *i*-a-Si:H layer in the *n^+^-n^-^* heterojunction has been removed. Both devices were measured under the same ambient conditions and at the same levels of illumination, illustrating the removal of the *i*-a-Si:H layer at the front has no detectable effect on the noise spectra in the frequency ranges explored in the paper. Unlike Fig. 3 of the paper, these spectra also include the dark data which was subtracted off, revealing the capacitive upturn. We note the difference between the devices manifests in this upturn, as described in Eq. 1 of the text.

­
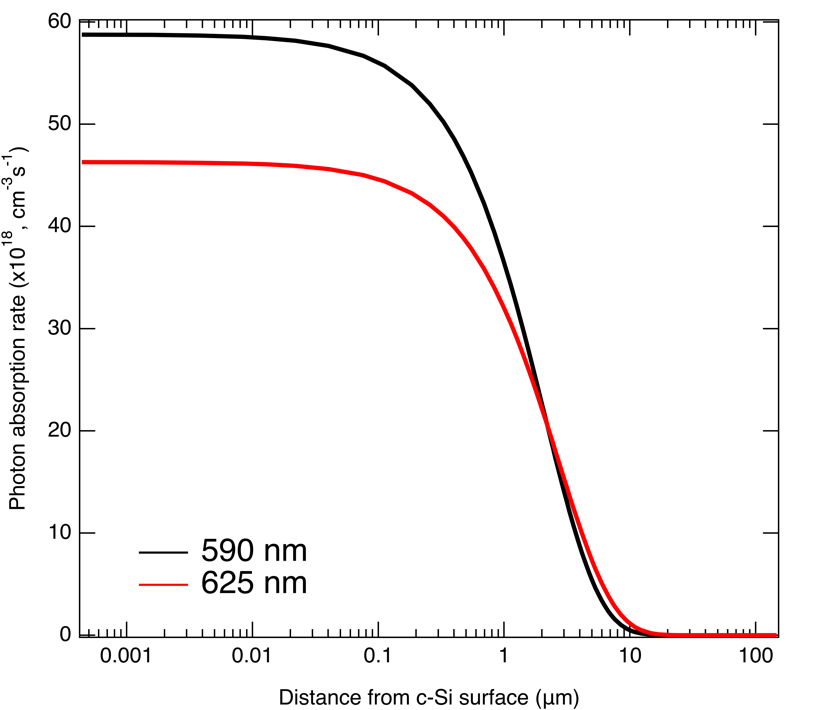


**Supplemental Figure S6.** Photon absorption rate as a function of distance from the surface of the c-Si wafer interface under both wavelengths. At a distance of ~10µm, this number goes to nearly zero, indicating that any light-dependent process must occur either in the front-facing layers or the first few tens of micrometers of the bulk wafer.


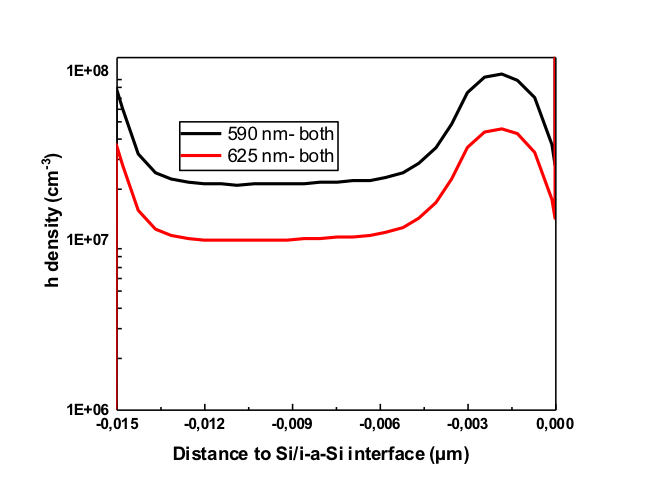


**Supplemental Figure S7. Top:** Simulated electron density in the n-a-Si/i-a-Si layer indicating a depletion towards the ITO interface; the zero point on the x axis represents the surface of the c-Si wafer. There is no observable difference in electron concentration with changing wavelength due to the fact that electron are majority carriers and are present in high concentration. **Bottom:** Simulated hole density in the n-a-Si/i-a-Si layer. There is a factor of three lower concentration of holes present under illumination for red light illumination, $\lambda=625 nm$ (red curve), as compared to the yellow light illumination, $\lambda=590 nm$ (black curve). The increase at the left side of the diagram indicates the effect of the partial depletion due to the ITO interface.


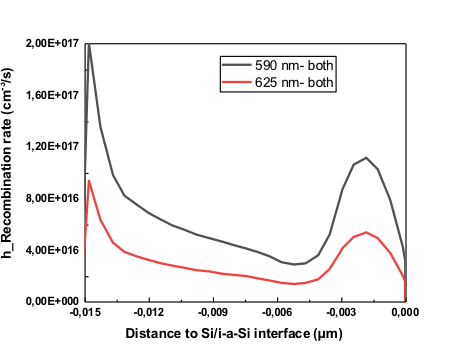


**Supplemental Figure S8.** Simulated hole recombination rate in the n-a-Si/i-a-Si layer; the zero point on the x axis represents the surface of the c-Si wafer. The increase on the left side indicates the enhanced interfacial recombination of holes due to the partial depletion near the ITO/n-a-Si interface.
